# Seasonal variation in haematological and biochemical reference values for healthy young children in The Gambia

**DOI:** 10.1186/s12887-016-0545-6

**Published:** 2016-01-11

**Authors:** Joseph Okebe, Julia Mwesigwa, Schadrac C. Agbla, Frank Sanya-Isijola, Ismaela Abubakar, Umberto D’Alessandro, Assan Jaye, Kalifa Bojang

**Affiliations:** Medical Research Council Unit, Fajara, Gambia; Institute of Tropical Medicine, Antwerp, Belgium; London school of Hygiene and Tropical Medicine, London, UK

**Keywords:** Reference values, Seasonality, Malaria, Haematological, Biochemical, Children

## Abstract

**Background:**

Haematological and biochemistry reference values for children are important for interpreting clinical and research results however, differences in demography and environment poses a challenge when comparing results. The study defines reference intervals for haematological and biochemistry parameters and examines the effect of seasonality in malaria transmission.

**Methods:**

Blood samples collected from clinically healthy children, aged 12–59 months, in two surveys during the dry and wet season in the Upper River region of The Gambia were processed and the data analysed to generate reference intervals based on the 2.5^th^ and 97.5^th^ percentiles of the data.

**Results:**

Analysis was based on data from 1141 children with median age of 32 months. The mean values for the total white cell count and differentials; lymphocyte, monocyte and neutrophil decreased with increasing age, were lower in males and higher in the wet season survey. However, platelet values declined with age (*p* < 0.0001). There was no evidence of effect of gender on mean values of AST, ALT, lymphocytes, monocytes, platelets and haemoglobin.

**Conclusion:**

Mean estimates for haematological and biochemistry reference intervals are affected by age and seasonality in the first five years of life. This consistency is important for harmonisation of reference values for clinical care and interpretation of trial results.

**Electronic supplementary material:**

The online version of this article (doi:10.1186/s12887-016-0545-6) contains supplementary material, which is available to authorized users.

## Background

Laboratory Reference ranges for haematological and biochemical parameters, derived from best available standards are used to guide eligibility into clinical trials, monitor participant safety, for external validity of results and to guide clinical management. Several research studies in sub-Saharan Africa (SSA) involve children less than five years and the import of haematological and biochemistry results obtained in these studies are inferred by comparing them against age-specific reference intervals [1–5]. Although useful, these reference values, derived using diverse methods, reflect the sampled population and may not account for environmental and genetic factors unique to SSA [6]. This is especially important in young children where nutrition and infections including malaria, play an important role in child health and development [3, 4]. This underscores the need for relevant context-specific [7, 8] studies that use standardised sampling and analytical methods in similar epidemiological setting to serve as reference [9].

Malaria significantly contributes to the morbidity especially anaemia in children less than five years. However, in the past decade, there has been a decline in malaria indices in several SSA countries including The Gambia [10, 11] with parasite prevalence as low as 9 % in a recent survey [12] . These reductions could imply a reduction in the prevalence of anaemia and a change in related haematological parameters. If this is the case, updates of reference values used for patient care and inclusion criteria into clinical trials are needed as well as establish baselines where these were previously unavailable.

This study describes laboratory reference intervals for clinically healthy children, aged 12–60 months, in The Gambia and assesses the effect of seasonality in malaria transmission on population-based haematological and biochemistry parameters.

## Methods

Two prospective cross sectional surveys were conducted in villages in the Upper River Region (URR) of The Gambia. The area is part of a health and demographic surveillance system (HDSS) which has records of births, deaths and movements updated quarterly [13]. Malaria transmission in the country is seasonal [14], lasting about six months (July-December) during and shortly after the rains and the surveys were carried out in September 2012 and May 2013; corresponding to the peak of the wet and dry seasons respectively.

Villages were selected by convenience sampling by location within a 10 km radius of the Medical Research Council (MRC) field station in Basse. This ensured that samples could be transferred and processed within two hours of collection. In each village, a random list of children aged 12 to 60 months, stratified by age; ≤30 months and >30 months, at the planned time of each survey was generated from the HDSS database. This stratification allowed for recruitment of similar proportions of children across each year strata. In each survey, a separate random list was generated to allow for changes in the demography in the villages due movement into or out of the area and entrance or exit from the target age range. A random selection approach was used because the aim was to produce population-level estimates and no attempts were made to exclude a child based on participation in the previous survey.

Following sensitization meetings, trained field staff comprising nurses and laboratory technicians visited the homes of selected children and obtained a written informed consent to participate from the parent or responsible guardian if the child was seen on the day of visit. The medical history taken by the team nurse focused on any episode of illness such as fever, frequent watery stools, and antibiotic use in the preceding two weeks, blood transfusions or any known medical condition such as sickle cell disease. A brief physical examination comprised of axillary temperature measurement, weight and height measurements, auscultation of the chest and abdominal palpation for enlarged spleen or liver was also done. Children with a documented temperature ≥37.5 °C at the time of visit, were screened for malaria using a rapid antigen-based text kit (RDT). Children with a positive RDT, although ineligible, were treated with an antimalarial or otherwise, referred to the nearest clinic for further care. Children who were eligible, had a venous blood sample collected in micro-EDTA (0.5mls) and heparinised (3.5mls) tubes and transported, in a portable cool box, to the field station for processing. Where a listed child was unavailable on the scheduled visit day, did not meet the eligibility criteria or caregiver did not consent, the next child on the list was identified and screened until the required sample target was reached.

A minimum of 120 samples for each parameter being evaluated is recommended to be able to derive a nonparametric 95 % reference intervals, allow for robust 90 % confidence limits for each reference limits after exclusion of inadequate or poor samples [7]. We sampled higher numbers of children to adjust for inadequate blood volume per child and errors from handling or processing.

### Laboratory analysis

Haematological parameters analysed included total white cell count (WBC_T_) and differentials: lymphocytes, monocytes, neutrophils and eosinophils, haemoglobin and platelets. Samples were analysed with a Quintus 5-part Haematology analyser (Boule Medical AB, Sweden). This uses impedance for measuring red and white blood cell components and spectrophotometry for haemoglobin measurement. The coefficient of variation for analytes are <1.8 % for white cell indices, <3.3 % for platelets and <1.0 % for haemoglobin. Samples were visually inspected for clotting or lysis and discarded if any was observed.

The biochemistry parameters analysed included sodium, potassium, urea, creatinine, aspartate aminotransferase (AST), alanine aminotransferase (ALT), total protein and albumin. Samples were centrifuged, serum separated and transferred to the main clinical laboratory in Fajara where they were processed using a VITROS 350 analyser (Ortho Clinical Diagnostics, USA). The VITROS 350 analyser is based on a dry slide chemistry technology. In summary, samples are placed on dry slides: a multi-layered analytical element coated on polyester supports containing appropriate substrate and other components needed for the reaction. The analyte catalyses the reaction sequence releasing products which absorb light at different wavelengths and the reflectance is converted to a quantifiable output. The coefficient of variation for analysis on the machine for samples are: sodium 0.9 %, potassium 1.6 %, urea 1.8 %, creatinine 2.5 %, AAST 2.4 %, ALT 11.2 %, total protein 1.1 and albumin 2.4 %. The laboratory has certification in good clinical laboratory practice (GCLP) and sample processing follows standard operating procedures developed at the laboratory. Calibrations of the analysers are done periodically, using positive and negative standards, based on the manufacturer’s instructions.

### Data cleaning and exclusion of outliers

Demographic and laboratory data were double entered on a database created using Microsoft Access (Microsoft Corp). The weight-for-height and height-for-age z-scores were determined using the World Health Organisation reference standards [15] and data from children with z-score below −3 SD were also excluded from the analysis. We fitted generalised additive models for location, scale and shape (GAMLSS) to account for possible influence of age, sex and season assessed the residuals on Q-Q plots to detect outliers. Outliers detected from residuals were excluded from the analysis (Table 1, Additional file 1: Table S1).

**Table 1:** Number of observations for each variable sample and the proportion included in the analysis

Variable	Total number of observations	Number of outliers identified	Observations (%) included in the analysis
Total WBC (10^9^/L)	1050	4	1046 (99.6)
Lymphocytes (10^9^/L)	1004	5	999 (99.5)
Monocytes (10^9^/L)	1003	2	1001 (99.8)
Neutrophils (10^9^/L)	1005	8	997 (99.2)
Eosinophils (10^9^/L)	1003	2	1001 (99.8)
Haemoglobin (g/dL)	1049	6	1043 (99.4)
Platelets (10^9^/L)	1060	20	1040 (98.1)
Sodium (mmol/L)	747	3	744 (99.6)
Potassium (mmol/L)	785	0	785 (100)
Urea (mmol/L)	798	4	794 (99.5)
Creatinine (μmmol/L)	794	5	789 (99.3)
AST (U/L)	779	15	764 (98.1)
ALT (U/L)	785	11	774 (98.6)
Albumin (g/L)	791	39	757 (95.7)

### Data analysis and determining reference intervals

Since all haematological and biochemistry variables were not normally distributed and classic nonparametric methods; log, square root transformations did not provide sufficiently normalised distribution. The variables were then modelled using GAMLSS with a Box-Cox power exponential (BCPE) distribution. The BCPE is suited for this type of analysis because it gives the flexibility to find a suitable transformation for different types of distributions because it allows for modelling four parameters: mean, standard deviation, skewness and kurtosis. Each of the four parameters were modelled as non-parametric smoothing cubic spline functions of age with optimal smoothing for each parameter were selected such that the generalized Akaike information criterion (AIC) was minimized with a penalty of three. This method has been clearly described and has been used in constructing growth curves such as the World Health Organisation reference standards [16–18]. We included seasonality and gender as explanatory variables in the model and constructed the 2.5^th^, 50^th^, and 97.5^th^ percentiles curves stratified by gender and/or seasonality if gender and/or seasonality were associated with the outcome variable. We assessed the model fit by examining the differences between observed and expected proportions of children below percentiles above mentioned and the residuals plots. Reference intervals are generated using the 2.5^th^ and 97.5^th^ centiles and presented as a summary and for each survey period. Reference intervals were also presented by age categories; 12 to 23, 24 to 35, 36 to 47 and 48 to 59 months. Data cleaning was done using Stata 12.1 (Stata Corp, TX) and the modelling performed using GAMLSS package in R software [17].

### Ethical approval

The Gambia Government/Medical Research Council Joint Ethics Committee approved the study (SCC 1298). The data generated in this manuscript is stored at the MRC Unit’s archive and is available on request from the authors.

## Results

Of the 1357 selected, 1261 children were enrolled in the study; 710 (56.3 %) during the rainy (malaria transmission) season. and 63 were excluded because they had documented temperature ≥37.5 °C, 3 had other symptoms but no fever. There were 54 severely malnourished children (weight-for-age z-score < −3SD); 30 and 24 in the wet and dry season respectively. In all, samples from 90.5 % (1141/1261) of enrolled children were used in the final analysis (Table 2). The median (IQR) age was 32 (22–45) months and 48 % (547/1141) were female. The median (IQR) weight and height were 11.6 kg (10–13.6) and 85.3 cm (78.2- 94.5) respectively. The number recruited and their gender was not significantly different between the season (*χ*^2^ = 0.063, *p* = 0.937). Children were less likely to be underweight (weight-for-height z-score < −2SD) during the wet season compared to the dry season (OR 0.51; 95%CI 0.34–0.77; *p* = 0.001). The proportion of children with moderate stunting (height-for-age z-scores < −2SD) was 34 % and was not significantly different between surveys.

**Table 2:** Baseline characteristics of study participants

	Characteristic	Number of children *N* = 1141 n (%)
Gender	Female	547 (47.9)
	Male	594 (52.1)
Season	Dry	518 (45.4)
	Rainy	623 (54.6)
Age group (months)	12–23	346 (30.3)
	24–25	329 (28.8)
	36–47	246 (21.6)
	47–59	220 (19.3)

### Reference intervals across seasons, age and gender

The reference intervals, based on the 2.5, 97.5^th^ percentiles, for haematology and biochemistry analytes are presented as a summary (Table 3) and stratified by age category, season and gender (Table 4, Additional file 2: Figure S1). The effect of the relationship exploratory variables; age, gender and seasonality on the mean values of the parameters analysed are also presented (Table 5). The mean values for the WBC_T_ and lymphocytes, monocytes and neutrophils decreased with increasing age, but higher in the wet season. In addition, the total WBC was lower in males (*p* = 0.01). Neutrophil was not associated with age (*p* = 0.13) but was lower in males (*p* = 0.004) and higher in the wet season (*p* = 0.001). The mean haemoglobin level increased with age (*p* < 0.0001) but was lower in the wet season survey. The reverse was seen with the mean platelet value which decreased with age (*p* < 0.0001), but were higher in the wet season (*p* < 0.0001). Sodium, urea, creatinine and albumin values increased with age with the estimated effect on the mean highest with creatinine (0.2) while potassium levels showed minimal but significant reduction with age (mean: −0.007; SE: 0.001; p < 0.0001), was lower in males (*p* = 0.02) and in the wet season (*p* = 0.002). There was no evidence of effect of gender on mean values of AST, ALT, lymphocytes, monocytes, platelets and haemoglobin.

**Table 3:** Reference intervals for haematological and biochemistry parameters in both dry and wet seasons

	All seasons		Dry season		Wet season	
Variable	N^a^	Reference interval^b^	N^a^	Reference interval^b^	N^a^	Reference interval^b^
Total WBC (10^9^/L)	1046	5.7–17.0	466	5.5–16.0	580	6.0–17.7
Lymphocytes (10^9^/L)	999	2.6–10.9	435	2.5–9.9	564	2.8–11.5
Monocytes (10^9^/L)	1001	0.08–1.30	434	0.10–1.21	567	0.07–1.34
Neutrophils (10^9^/L)	997	1.3–6.9	434	1.3–6.3	563	1.3–7.3
Eosinophils (10^9^/L)	1001	0.06–1.59	434	0.06–1.64	567	0.05–1.54
Haemoglobin (g/dL)	1043	7.2–13.1	463	7.3–13.6	580	7.1–12.4
Platelets (10^9^/L)	1040	164.2–809.3	462	142.1–772.3	578	188.3–831.7
Sodium (mmol/L)	744	133.8–144.8	420	135.2–145.5	324	131.9–143.4
Potassium (mmol/L)	785	3.8–6.2	428	3.9–6.2	357	3.7–6.2
Urea (mmol/L)	793	0.9–4.4	438	1.0–4.4	355	0.8–4.3
Creatinine (μmmol/L)	789	8.8–37.9	433	13.7–39.7	356	7.8–27.6
AST U/L	764	20.4–62.3	423	26.6–61.7	341	16.6–64.1
ALT U/L	774	9.3–36.4	425	8.7–29.2	349	10.8–39.8
Albumin g/dL	766	25.9–47.1	430	30.9–47.4	336	23.8–45.7

**Table 4:** Median and reference intervals for haematology and biochemistry values stratified by season, by age group and/or by sex

		Dry season				Wet season			
		Median (reference interval)^a^				Median (reference interval) ^a^			
		12–23 months	24–35 months	36–47 months	48–59 months	12–23 months	24–35 months	36–47 months	48–59 months
Total WBC (10^9^/L)	Female	11.3 (6.2–16.9)	10.1 (6.3–16.5)	8.6 (5.5–14.9)	8.5 (5.4–14.7)	11.6 (6.4–20.8)	10.4 (6.3–17.7)	9.3 (6.0–15.2)	8.8 (6.1–13.7)
	Male	10.2 (6.0–15.7)	9.8 (5.9–15.8)	8.8 (5.3–14.8)	7.8 (4.6–14.2)	11.3 (6.5–19.2)	10.0 (6.2–17.2)	9.1 (5.9–15.3)	8.6 (5.7–13.8)
Lymphocytes (10^9^/L)	All gender	6.3 (3.0–10.5)	5.7 (2.8–10.1)	4.5 (2.3–8.3)	4.2 (2.3–8.1)	6.8 (3.5–13.5)	5.6 (3.0–10.7)	4.9 (2.6–8.8)	4.6 (2.6–8.1)
Monocytes (10^9^/L)	All gender	0.49 (0.10–1.37)	0.44 (0.10–1.24)	0.40 (0.09–1.10)	0.35 (0.09–0.94)	0.61 (0.09–1.46)	0.55 (0.08–1.34)	0.49 (0.06–1.23)	0.44 (0.05–1.11)
Neutrophils (10^9^/L)	Female	3.2 (1.3–6.8)	3.1 (1.3–7.0)	3.0 (1.2–7.0)	3.0 (1.3–6.7)	3.3 (1.4–7.9)	3.3 (1.4–7.3)	3.2 (1.5–6.8)	3.1 (1.5–6.3)
	Male	2.9 (1.4–6.2)	2.9 (1.3–5.7)	2.8 (1.3–5.4)	2.8 (1.2–5.1)	3.1 (1.3–8.5)	3.0 (1.4–7.6)	3.0 (1.3–6.5)	3.0 (1.2–5.6)
Eosinophils (10^9^/L)	All gender	0.32 (0.05–1.31)	0.36 (0.06–1.49)	0.40 (0.06–1.69)	0.43 (0.06–1.90)	0.32 (0.05–1.31)	0.36 (0.06–1.49)	0.40 (0.06–1.69)	0.43 (0.06–1.90)
Haemoglobin (g/dL)	All gender	10.2 (6.8–12.7)	10.7 (7.3–13.3)	11.2 (7.8–13.8)	11.7 (8.4–14.2)	9.6 (7.2–11.6)	10.1 (6.9–12.0)	10.8 (7.2–12.5)	11.1 (7.6–12.7)
Platelets (10^9^/L)	All gender	478.8 (146.1–885.8)	433.6 (159.2–737.4)	397.8 (143.4–665.3)	375.7 (125.9–632.0)	530.9 (162.9–921.5)	487.6 (182.6–817.0)	449.9 (200.0–720.2)	421.8 (219.7–641.6)
Sodium (mmol/L)	All gender	140.1 (134.3–143.9)	140.4 (135.2–144.7)	140.6 (135.8–145.9)	140.9 (136.2–148.0)	139.1 (131.4–142.8)	139.5 (132.0–143.2)	140.0 (132.6–143.6)	140.4 (133.3–144.0)
Potassium (mmol/L)	Female	5.1 (4.0–6.5)	5.0 (3.9–6.4)	5.0 (3.8–6.2)	4.9 (3.7–6.1)	5.0 (4.0–6.3)	4.9 (3.2–5.9)	4.8 (3.7–6.8)	4.6 (3.9–6.2)
	Male	5.0 (4.0–6.1)	4.9 (3.9–6.1)	4.8 (3.8–6.1)	4.8 (3.8–6.1)	4.9 (3.7–6.2)	4.9 (3.8–6.1)	4.8 (3.9–5.8)	4.6 (3.8–5.3)
Urea (mmol/L)	Female	1.9 (0.8–4.2)	2.5 (1.1–4.4)	2.7 (1.4–4.3)	2.7 (1.7–4.0)	1.5 (0.6–3.4)	1.9 (0.9–3.8)	2.4 (1.2–4.2)	2.8 (1.5–4.7)
	Male	2.0 (0.8–3.8)	2.4 (1.1–4.1)	2.7 (1.5–4.3)	2.9 (1.8–4.3)	1.7 (0.7–3.3)	2.2 (1.0–4.1)	2.5 (1.1–4.6)	2.7 (1.2–4.8)
Creatinine (μmmol/L)	Female	25.8 (12.9–33.0)	27.6 (13..5–37.9)	30.0 (17.5–40.0)	32.5 (17.6–43.3)	12.4 (6.7–23.2)	15.5 (8.5–24.4)	18.7 (8.5–28.3)	21.7 (15.0–27.1)
	Male	26.4 (17.0–40.7)	28.3 (15.4–41.6)	29.7 (14.8–40.5)	31.3 (22.7–38.9)	13.8 (6.7–24.0)	16.3 (8.2–25.3)	18.8 (9.9–27.0)	21.2 (11.9–28.7)
AST (U/L)	All gender	36.5 (29.2–61.8)	38.9 (27.4–62.1)	38.3 (25.9–61.7)	37.7 (24.5–61.0)	37.2 (17.3–64.7)	37.9 (17.2–65.2)	37.6 (16.7–64.3)	35.1 (15.2–59.4)
ALT (U/L)	All gender	16.5 (8.2–29.8)	16.3 (8.7–29.8)	16.1 (9.2–29.7)	15.9 (9.6–29.6)	20.4 (9.9–39.0)	21.8 (10.8–39.6)	22.9 (11.6–39.8)	23.6 (12.1–39.8)
Albumin (g/L)	All gender	39.4 (31.2–46.4)	39.8 (31.0–47.0)	40.3 (30.9–47.6)	40.8 (30.9–48.4)	36.8 (21.4–45.5)	37.5 (23.1-45.6)	38.3 (24.8-45.7)	39.0 (26.6–45.9)

^a^The 2.5 and 97.5 percentiles are used to define the reference interval

**Table 5:** Association between all haematology and biochemistry parameters and explanatory variables age, sex and season

Parameter	Estimated effect on the median (SE)^a^ value of parameter		
	Cubic spline of age^b^	Gender^c^	Season^d^
Total WBC (10^9^/L)	−0.08 (0.006); *p* < 0.0001	−0.07 (0.15); *p* = 0.01	0.56 (0.15); *p* = 0.0002
Lymphocytes (10^9^/L)	−0.06 (0.004); *p* < 0.0001	−0.05 (0.10); *p* = 0.65	0.31 (0.10); *p* = 0.004
Monocytes (10^9^/L)	−0.004 (0.001); *p* < 0.0001	−0.02 (0.02); *p* = 0.16	0.06 (0.02); *p* = 0.0002
Neutrophils (10^9^/L)	−0.004 (0.003); *p* = 0.13	−0.22 (0.08); *p* = 0.004	0.26 (0.08); *p* = 0.0007
Eosinophils (10^9^/L)	0.003 (0.001); *p* < 0.0001	0.02 (0.02); *p* = 0.31	−0.02 (0.02); *p* = 0.24
Haemoglobin (g/dL)	0.041 (0.002); *p* < 0.0001	−0.053 (0.068); *p* = 0.44	−0.662 (0.069); *p* < 0.0001
Platelets (10^9^/L)	−2.75 (0.29); *p* < 0.0001	−8.34 (7.43); *p* = 0.26	44.0 (7.41); *p* < 0.0001
Sodium (mmol/L)	0.03 (0.01); *p* < 0.0001	−0.24 (0.16); *p* = 0.13	−0.92 (0.16); *p* < 0.0001
Potassium (mmol/L)	−0.007 (0.001); *p* < 0.0001	−0.09 (0.04); *p* = 0.02	−0.12 (0.04); *p* = 0.002
Urea (mmol/L)	0.03 (0.002); *p* < 0.0001	0.12 (0.05); *p* = 0.02	−0.19 (0.05); *p* = 0.003
Creatinine (μmol/L)	0.20 (0.01); *p* < 0.0001	0.77 (0.29); *p* = 0.01	−10.9 (0.35); *p* < 0.0001
AST (U/L)	−0.07 (0.02); *p* = 0.005	0.44 (0.65); *p* = 0.50	−1.88 (0.65); *p* = 0.004
ALT (U/L)	0.03 (0.01); *p* = 0.02	−0.09 (0.37); *p* = 0.80	5.80 (0.40); p < 0.0001
Albumin g/dL	0.04 (0.01); *p* < 0.0001	−0.02 (0.26); *p* = 0.39	−1.93 (0.26); *p* < 0.0001

^a^ Standard errors of the estimated effect on the median value

^b^ A positive sign of the coefficient indicates that the median value increases with age

^c^ Males are compared to females (reference)

^d^ Wet season is compared to dry season (reference)

## Discussion

This study evaluated laboratory reference values among children less than five years, who bear the greatest burden of morbidity and mortality in developing countries and, are enrolled in clinical trials. Clinical and asymptomatic malaria is significantly associated with the prevalence of anaemia therefore clinical studies apply cut-off value for haemoglobin to minimise any effect of confounding by anaemia and infections.

Haematological and biochemistry parameters showed strong associations with age and seasonality but not gender. Of note is that a decrease in the mean values for the platelets, WBC_T_ and the assessed differentials (except eosinophils) with increasing age category but with higher mean values in the wet season. However, haemoglobin values increased with age. The effect of age on the mean estimate for electrolytes was less consistent with mean sodium, urea, creatinine and albumin increasing with age and most electrolytes being lower in the wet season compared with the dry season.

When compared to reference intervals generated for infants, we observe that the reference intervals for haemoglobin, WBC_T_ and differentials are comparable; the main difference being that those from this study but with slightly lower limits and wider range of values compared to infants and data from western countries compared in the paper [19]. The consistency of the data is also seen when compared to reference intervals for older children and adults which also showed similar trends for WBCs and haemoglobin and no association with gender [20]. This means that the results are indeed comparable and applicable across the country and in settings where trends of malaria transmission are similar. Between-season variations are seen with platelets, creatinine and the liver enzymes; AST and ALT where there is a shift in the range of the intervals towards higher values in the wet season in all except ALT which the effect was reversed. Although these children were clinically well, these higher ranges may be due to low grade inflammation possibly from or subclinical malaria and/ or bacteraemic infections during the wet season [21].

Nutritional deficiency is an important contributor to the risk of morbidity and seasonal fluctuations in the quality, quantity and range of available diet of children does play a role in susceptibility to infections [22–24]. Severely malnourished children were excluded from the analysis since the aim of the study was not to describe changes due to severe malnutrition however, these reference intervals would be useful in monitoring progress with rehabilitation of malnourished patients.

A comparison of biochemistry reference with other studies showed lower values for AST but not for potassium or ALT with higher values of liver enzymes are noted in infants which decline towards adulthood [19]. Reference intervals for creatinine and sodium were lower than reported in western settings [8] and show an increase with age.

There was no observed association with gender and haematological and biochemistry parameters which would suggest that gender-based differences in these parameters may be due to sex-hormones [20, 25] which is unlikely in this population.

The analytical approach applied in the study took into account the need to derive suitably normally distributed data to estimate the reference range which was not possible using classic data transformations. The GAMLSS offers a great flexibility and with the BCPE transformation, we include the skewness and kurtosis in normalizing the data. This is evident in its growing application in establishing reference intervals such as the WHO reference standards [18].

Overall, the study shows that the mean values for some haematological and biochemical parameters are affected by age and seasonality. However, the reference intervals are broadly consistent. This should improve confidence in their use in clinical care and research.

## Conclusion

Reference intervals for haematological indices show significant statistical differences by age and seasonality in the first five years of life. Biochemistry parameters may be less variable but the results are consistent with reference intervals generated from other parts of the country using different methods. These variations could be important in statistical inference but maybe less so in clinical care.

## Additional files

Additional file 1: Table S1. Proportions of observations below predicted percentiles from models with and without outliers. (DOC 64 kb) Additional file 2: Figure S1. Median and reference intervals (2.5^th^-97.5^th^) for all haematology and biochemistry parameters over age, by gender and/or season. (PDF 1045 kb)

## Abbreviations

ALT: Alanine aminotransferase
AST: Aspartate aminotransferase
BCPE: Box-Cox power exponential
GAIC: Generalised Akaike information criterion
CI: Confidence interval
EDTA: Ethylenediaminetetraacetic acid
GAMLSS: Generalised additive models for location scale and shape ()
GCLP: Good clinical laboratory practice
HDSS: Health and demographic surveillance system
IQR: Inter-quartile range
MRC: Medical Research Council Unit The Gambia
RDT: Rapid diagnostic test
SD: Standard deviation
SSA: Sub-Saharan Africa
URR: Upper river region
WBC_T_: Total white blood cell count
