# Supplementary material for: Seasonal variation in haematological and biochemical reference values for healthy young children in The Gambia
Source: BMC Pediatr. 2016 Jan 11;16:5. doi: 10.1186/s12887-016-0545-6 (PMC4710011; doi:10.1186/s12887-016-0545-6)
Supplement: Additional file 1: Table S1. — Proportions of observations below predicted percentiles from models with and without outliers. (DOC 64 kb) [file 12887_2016_545_MOESM1_ESM.doc]

| Haematology and biochemistry parameters | Percentiles | Proportions of observations below predicted percentiles (outliers included) | Proportions of observations below predicted percentile (outliers excluded) |
| --- | --- | --- | --- |
| Total WBC (10^9^/L) | 2.5 | 1.0 | 2.8 |
|  | 50 | 50.9 | 49.1 |
|  | 97.5 | 96.7 | 97.1 |
| Lymphocytes (10^9^/L) | 2.5 | 1.5 | 2.6 |
|  | 50 | 49.5 | 49.2 |
|  | 97.5 | 97.6 | 97.6 |
| Monocytes (10^9^/L) | 2.5 | 2.7 | 2.8 |
|  | 50 | 48.3 | 48.0 |
|  | 97.5 | 98.5 | 97.1 |
| Neutrophils (10^9^/L) | 2.5 | 1.8 | 2.6 |
|  | 50 | 50.1 | 49.3 |
|  | 97.5 | 96.9 | 97.1 |
| Eosinophils (10^9^/L) | 2.5 | 2.5 | 2.5 |
|  | 50 | 50.6 | 50.7 |
|  | 97.5 | 97.0 | 97.1 |
| Haemoglobin (g/dL) | 2.5 | 3.0 | 2.7 |
|  | 50 | 48.3 | 48.6 |
|  | 97.5 | 97.4 | 97.5 |
| Platelets (10^9^/L) | 2.5 | 4.0 | 3.0 |
|  | 50 | 49.9 | 50.3 |
|  | 97.5 | 98.0 | 97.4 |
| Sodium (mmol/L) | 2.5 | 2.8 | 2.6 |
|  | 50 | 50.5 | 50.9 |
|  | 97.5 | 97.5 | 97.2 |
| Potassium (mmol/L)^a^ | 2.5 | 2.5 | 2.5 |
|  | 50 | 50.3 | 50.3 |
|  | 97.5 | 97.3 | 97.3 |
| Urea (mmol/L) | 2.5 | 3.1 | 2.8 |
|  | 50 | 51.1 | 50.8 |
|  | 97.5 | 97.5 | 97.4 |
| Creatinine (µmmol/L) | 2.5 | 2.8 | 2.5 |
|  | 50 | 48.7 | 48.9 |
|  | 97.5 | 98.1 | 97.1 |
| AST (U/L) | 2.5 | 3.3 | 2.5 |
|  | 50 | 49.8 | 49.7 |
|  | 97.5 | 96.7 | 96.9 |
| ALT (U/L) | 2.5 | 2.8 | 2.8 |
|  | 50 | 49.8 | 49.5 |
|  | 97.5 | 97.5 | 97.3 |
| Albumin (g/L) | 2.5 | 7.1 | 3.8 |
|  | 50 | 50.9 | 51.0 |
|  | 97.5 | 99.1 | 97.7 |

^a^ No outliers were identified.
