# Supplementary material for: Seasonal variation in haematological and biochemical reference values for healthy young children in The Gambia
Source: BMC Pediatr. 2016 Jan 11;16:5. doi: 10.1186/s12887-016-0545-6 (PMC4710011; doi:10.1186/s12887-016-0545-6)
Supplement: Additional file 2: Figure S1. — Median and reference intervals (2.5th-97.5th) for all haematology and biochemistry parameters over age, by gender and/or season. (PDF 1045 kb) [file 12887_2016_545_MOESM2_ESM.pdf]

A. WBC (red curve: median, blue curve: 2.5th and black curve: 97.5th)

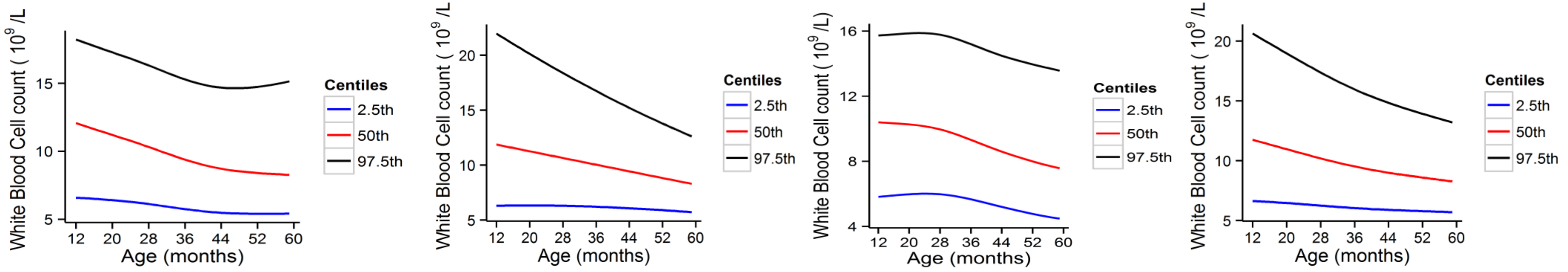

Female in dry season | Female in wet season | Male in dry season | Male in wet season

B. Lymphocyte (red curve: median, blue curve: 2.5th and black curve: 97.5th)

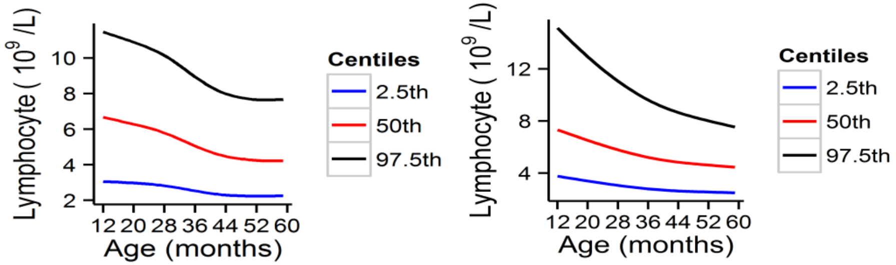

In dry season | In wet season

C. Monocyte (red curve: median, blue curve: 2.5th and black curve: 97.5th)

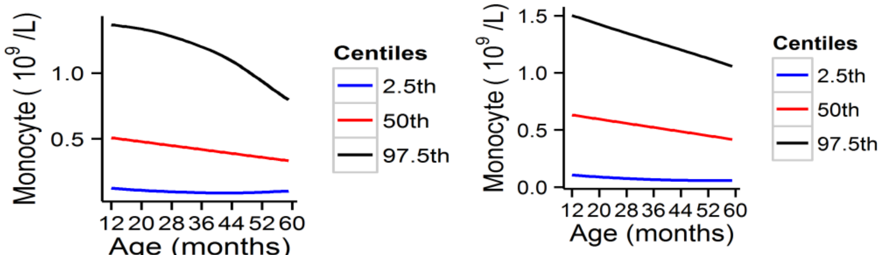

In dry season | In wet season

D. Neutrophils (red curve: median, blue curve: 2.5th and black curve: 97.5th)

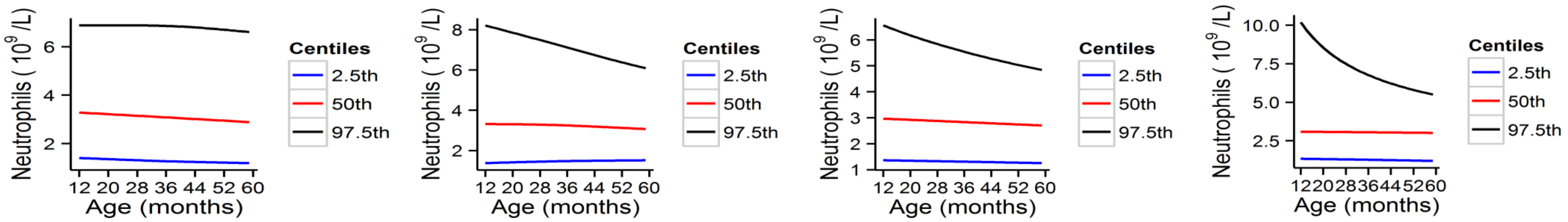

Female in dry season | Female in wet season | Male in dry season | Male in wet season

E. Eosinophils (red curve: median, blue curve: 2.5th and black curve: 97.5th, all seasons and gender)

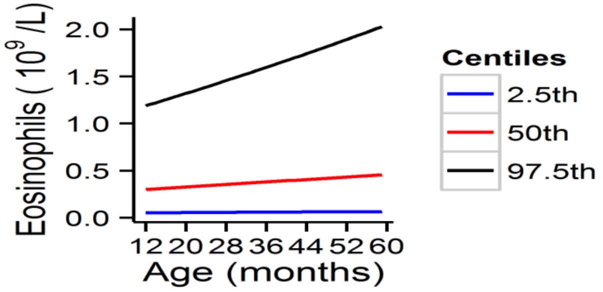

All seasons and gender

F. Haemoglobin (red curve: median, blue curve: 2.5th and black curve: 97.5th)

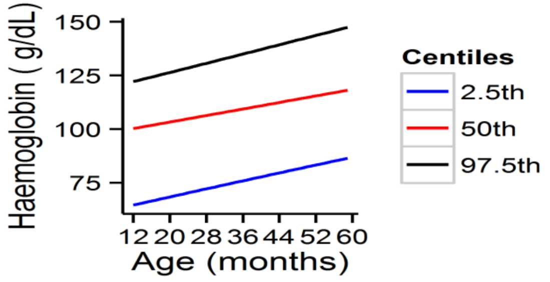

In dry season

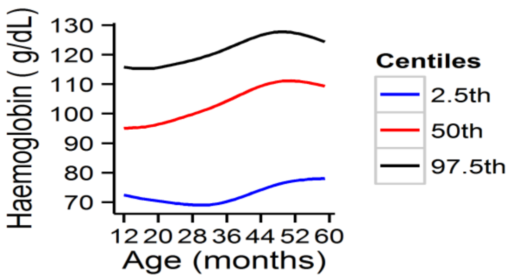

In wet season

G. Platelets (red curve: median, blue curve: 2.5th and black curve: 97.5th)

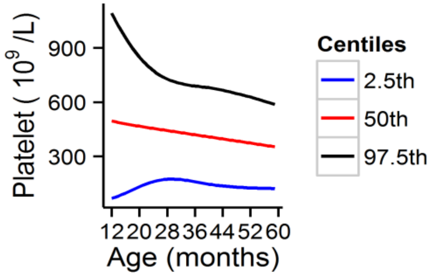

In dry season

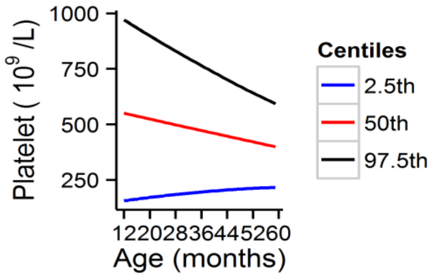

In wet season

H. Sodium (red curve: median, blue curve: 2.5th and black curve: 97.5th)

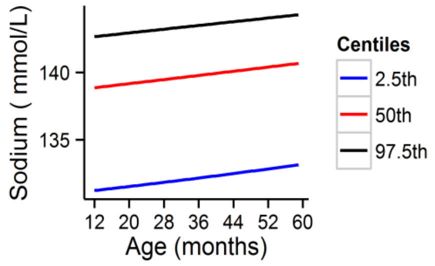

In dry season

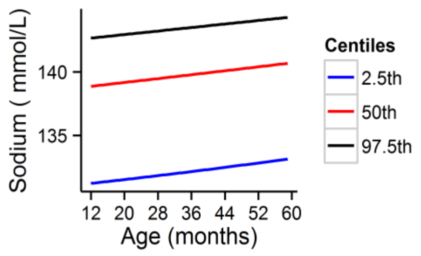

In wet season

I. Potassium (red curve: median, blue curve: 2.5th and black curve: 97.5th)

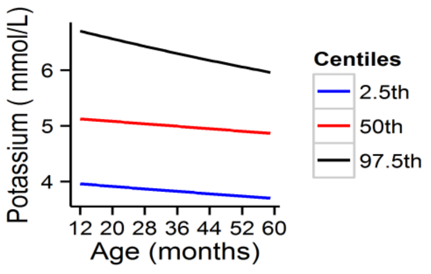

Female in dry season

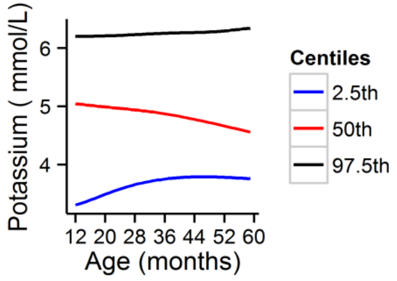

Female in wet season

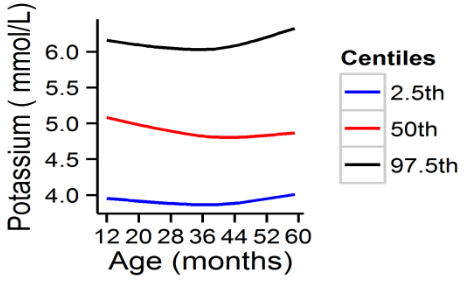

Male in dry season

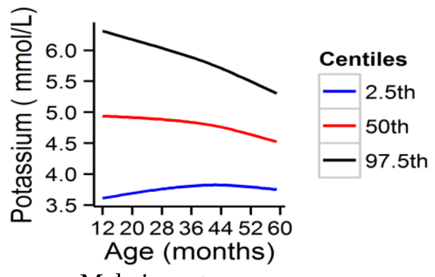

Male in wet season

J. Urea (red curve: median, blue curve: 2.5th and black curve: 97.5th)

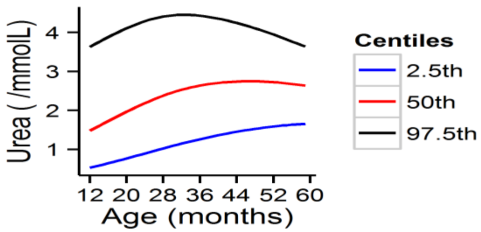

Female in dry season

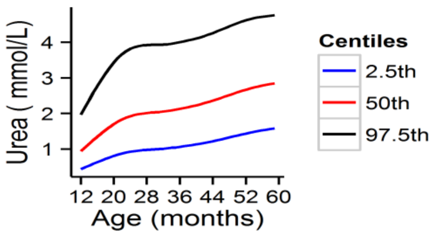

Female in wet season

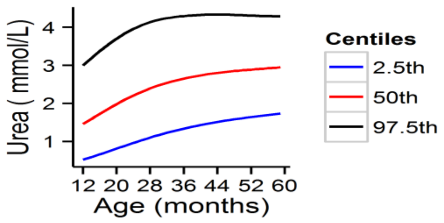

Male in dry season

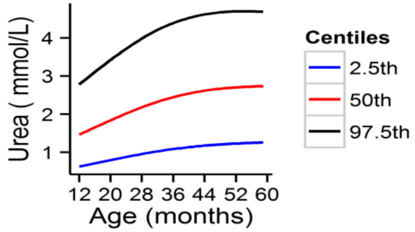

Male in wet season

K. Creatinine (red curve: median, blue curve: 2.5th and black curve: 97.5th)

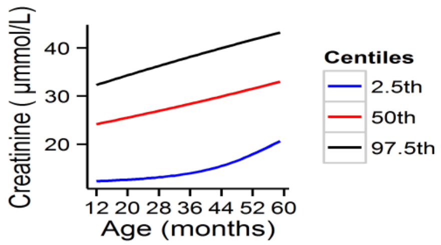

Female in wet season

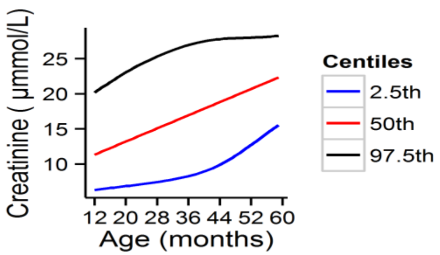

Male in dry season

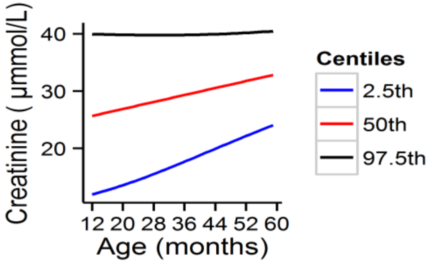

Male in wet season

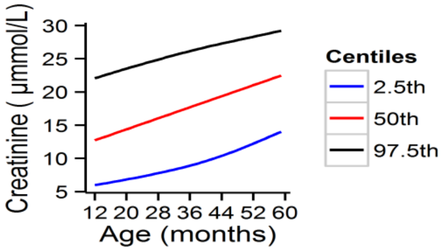

Male in wet season

L. AST (red curve: median, blue curve: 2.5th and black curve: 97.5th)

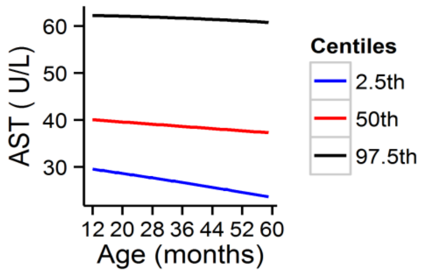

In dry season

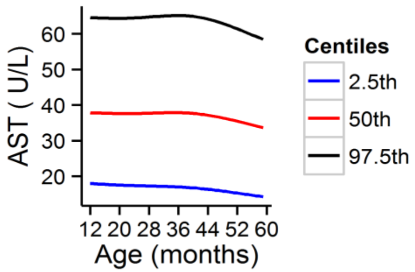

In wet season

M. ALT (red curve: median, blue curve: 2.5th and black curve: 97.5th)

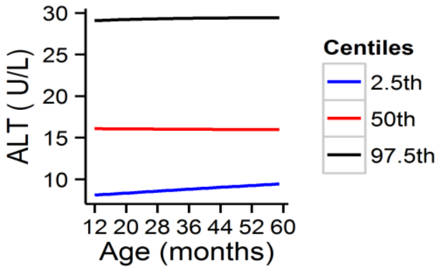

In dry season

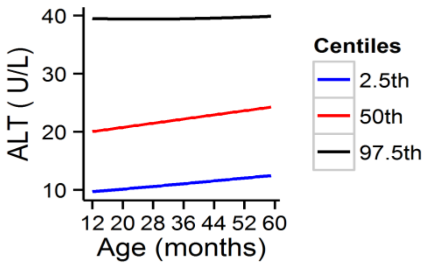

In wet season

N. Albumin (red curve: median, blue curve: 2.5th and black curve: 97.5th)

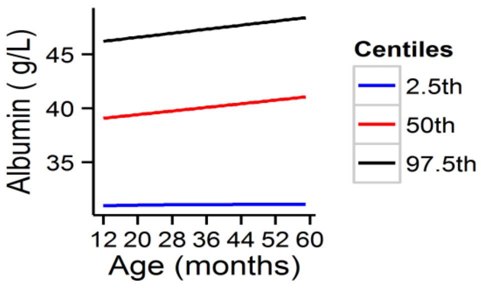

In dry season

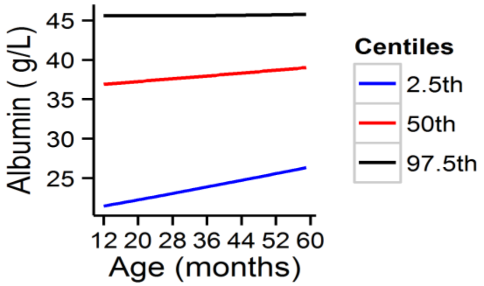

In wet season
